# Genetic Characteristics of Cutaneous, Acral, and Mucosal Melanoma in Japan

**DOI:** 10.1002/cam4.70360

**Published:** 2024-11-20

**Authors:** Tokimasa Hida, Masashi Idogawa, Junji Kato, Yukiko Kiniwa, Kohei Horimoto, Sayuri Sato, Masahide Sawada, Shoichiro Tange, Masae Okura, Ryuhei Okuyama, Takashi Tokino, Hisashi Uhara

**Affiliations:** ^1^ Department of Dermatology Sapporo Medical University School of Medicine Sapporo Japan; ^2^ Department of Medical Genome Sciences Cancer Research Institute, Sapporo Medical University School of Medicine Sapporo Japan; ^3^ Department of Dermatology Shinshu University School of Medicine Nagano Japan

**Keywords:** DNA copy number variation, immune checkpoint inhibitor, melanoma, molecular targeted therapy, mutation

## Abstract

**Background:**

Acral and mucosal melanomas are more prevalent in Asians than in Caucasians, unlike cutaneous melanomas, which are predominant in Caucasians. Recent studies have suggested that non‐Caucasian cutaneous melanomas responded less to immune checkpoint inhibitors, highlighting the need for genetic profiling across ethnicities. This study aimed to elucidate the genetic characteristics of Japanese melanomas, which is an under‐researched topic.

**Methods:**

Single‐nucleotide variants, indels, and copy number alterations in 104 Japanese melanoma patients (37 cutaneous, 52 acral, and 15 mucosal) were analyzed using custom panel sequencing.

**Results:**

Driver events were detected in 94% of the cases. Among cutaneous melanoma cases, 76% had BRAF mutations, and 8% had NRAS mutations. In acral melanoma, BRAF (9%), NRAS (17%), KRAS (8%), KIT (19%), and NF1 (7%) mutations were detected. Major driver mutations in mucosal melanoma were detected in NRAS, KRAS, NF1, PTEN, GNAQ, and KIT. The median tumor mutational burden across all melanoma types was 4.6 mutations/Mb, with no significant difference between the cutaneous and acral/mucosal types. Of the 21 patients with both primary and metastatic lesions, 11 showed distinct mutations in each. Potentially actionable mutations were detected in 58 patients in addition to BRAF V600E/K mutations in 31.

**Conclusions:**

This study highlights distinct genetic abnormalities and actionable alterations in Japanese melanoma patients. This suggests a lower tumor mutational burden in East Asian cutaneous melanoma, which may affect the efficacy of immune checkpoint inhibitors. The heterogeneity of driver mutations across and within individuals highlights the need for personalized treatment approaches.

## Graphical abstract

Single‐nucleotide variants, indels, and copy number alterations in 104 Japanese melanoma patients were analyzed using custom panel sequencing. This study highlights distinct genetic abnormalities and actionable alterations in Japanese melanoma patients. Importantly, Japanese patients with cutaneous melanoma exhibited a lower tumor mutational burden than that reported in Caucasian patients, which may affect the efficacy of immune checkpoint inhibitors.

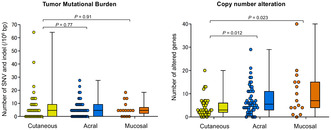

## 1 Introduction

High‐throughput sequencing technologies have revolutionized melanoma genome profiling, revealing extensive genetic alterations and characterizing melanoma as a heterogeneous group of disorders. The Cancer Genome Atlas (TCGA) project has identified distinct subtypes of cutaneous melanoma (CM), primarily defined by mutations in *BRAF*, *RAS*, *NF1*, and triple wild‐type subtypes [1]. This classification has been crucial in understanding the genetic drivers of CM and in developing targeted therapies. In contrast to TCGA, the WHO's 2018 and 2023 classifications encompass all melanoma subtypes, including acral melanoma (AM), mucosal melanoma (MM), and various CM subtypes [2, 3]. This comprehensive approach is crucial for grasping the genetic differences among these subtypes, particularly the rarer AM and MM.

UV radiation significantly impacts the tumor mutational burden (TMB) in CM, leading to a significantly high TMB in this subtype [4]. Desmoplastic melanoma, within CM subtypes, exhibits the highest TMB, followed by high‐cumulative sun damage (CSD) and low‐CSD melanomas [2]. AM and MM, minimally affected or unaffected by UV, have lower TMB but a higher prevalence of structural variants and copy number alterations (CNAs), suggesting a distinct pathogenesis from CM [4, 5, 6].

Advances in pharmacological treatments of melanoma have paralleled genetic research. Selective BRAF inhibitor monotherapy and BRAF/MEK inhibitor combinations are more effective than traditional chemotherapy in treating *BRAF*‐mutated melanoma [7, 8, 9]. Studies in Western countries have shown that immune checkpoint inhibitors (ICIs), including an anti‐CTLA‐4 antibody (ipilimumab) and anti‐PD‐1 antibodies (nivolumab and pembrolizumab), are effective in advanced melanoma, especially in CM with high TMB [10, 11, 12]. However, challenges such as limited patient response to ICIs in AM and MM and acquired resistance to BRAF/MEK inhibitors persist [13, 14].

Ethnic differences in melanoma subtypes and treatment responses were also noted. Caucasians predominantly have CM, while non‐Caucasians such as East Asians, Hispanics, and Africans more often develop AM and MM [15]. Previous genetic studies in Western countries have mainly included Caucasian patients for CM, with non‐Caucasians better represented in AM and MM groups [5, 16]. This racial disparity in research implies potential racial biases in the reported genetic differences among melanoma subtypes. While most clinical trials have focused on Caucasian patients, the efficacy of ICIs in non‐Caucasians remains unclear [13]. Recent studies suggest that ethnicity‐specific factors, in addition to melanoma subtypes, influence the response to immunotherapy [15, 17]. For instance, TMB in CM may vary across ethnicities; however, comprehensive data for non‐Caucasians are lacking. Furthermore, targeted therapies other than BRAF/MEK inhibitors are needed, particularly for ethnic groups with a lower incidence of *BRAF*‐mutated melanoma. In East Asian populations, the genetic landscape of melanoma is under‐researched. In Japan, a single study has detailed the prevalence of hotspot mutations in *BRAF*, *NRAS*, and *KIT* [18].

To explore the genetic landscape of melanoma in Japan and identify new therapeutic options, we developed a custom melanoma‐specific sequencing panel. Analysis of 104 Japanese melanoma patients facilitated a comparison of genetic profiles between the Japanese and other ethnicities, highlighting the unique genetic landscape of melanoma in Japan. Importantly, Japanese patients with CM exhibited a lower TMB than that reported in Caucasian patients. Furthermore, numerous actionable mutations that are typically undetectable in routine clinical practice were identified. These findings highlight the potential of this research to guide more effective and personalized melanoma treatment.

## 2 Materials and Methods

### 2.1 Sample Acquisition and DNA Extraction

Patients were enrolled at Sapporo Medical University Hospital and Shinshu University Hospital between 2020 and 2023. Primary and/or metastatic tumor tissues were collected from each patient. Sample preparation and sequencing methods have been previously described [19, 20]. Briefly, tumor tissues were manually dissected from unstained formalin‐fixed paraffin‐embedded (FFPE) sections, each 10 μm thick. The QIAamp DNA FFPE Tissue Kit (Qiagen, Valencia, CA) was used for DNA extraction from the tissue samples. When fresh‐frozen tissue was available, DNA was extracted using a DNA Extractor kit (Fujifilm Wako Pure Chemical Co. Osaka, Japan). If the DNA eluate contained visible melanin, it was removed using the OneStep PCR Inhibitor Removal Kit (Zymo Research, Irvine, CA). DNA from normal tissues, such as peripheral blood or saliva, was extracted using a DNA Extractor WB kit (Fujifilm Wako Pure Chemical Co.) for germline variant detection. In archival FFPE cases where normal blood or saliva could not be sourced, DNA was extracted from the normal tissue within the FFPE specimens.

### 2.2 Library Preparation and Targeted Sequencing

For library preparation, 100 ng of DNA was amplified by multiplex PCR using the Ion AmpliSeq Library Kit Plus (Thermo Fisher Scientific) and a custom melanoma panel designed by the Ion AmpliSeq Designer (Thermo Fisher Scientific). The panel comprised 3336 primer pairs for 95 genes identified as drivers or significantly mutated genes as of May 2020 (Table S1) [1, 4, 5, 21, 22]. The panel had previously been used to analyze a benign skin tumor [23]. Libraries were prepared following the manufacturer's instructions. For sequencing, we used the Ion GeneStudio S5 system (Thermo Fisher Scientific) following the established protocol. The templates were subjected sequencing post‐emulsion PCR using the Ion 540 Chip Kit and Ion 540 Kit‐Chef (Thermo Fisher Scientific).

### 2.3 Detection of Single‐Nucleotide Variants (SNVs) and Indels

Sequencing data in FASTQ format were aligned to the hg38 human genome reference using the Burrows–Wheeler Aligner tool [24]. The resulting SAM files were sorted and converted to BAM format, as previously described [25, 26]. Somatic SNVs and indels were identified using Varscan2 by comparing tumor and normal tissue genomic alterations [27]. ANNOVAR was used to annotate amino acid substitutions [28]. All identified mutations were visualized using the Integrative Genomics Viewer to rule out strand bias, homopolymers, and mispriming [29]. Mutations were included if they had a frequency of ≥ 10%, were absent or present at < 1% in normal controls, and had at least three mutant reads in the tumor sample. Mutations with an allele frequency of 5% or more, but less than 10%, were included after confirming their validity. For the *TERT* promoter region, we examined nucleotide mutations at positions −57, −124, −138, and −146 from the ATG site. Due to the difficulty in amplifying the GC‐rich region by PCR, we established lenient criteria for mutation identification: A mutation must be present in at least two reads in the tumor sample and absent in the normal sample. The pathogenicity of somatic mutations was assessed using the ClinVar and the Catalog of Somatic Mutations in Cancer (COSMIC, https://cancer.sanger.ac.uk/cosmic, accessed on November 1, 2023) databases [30, 31]. SNVs and indels classified as Tier 1–3 in the COSMIC Cancer Mutation Census, along with frameshift, nonsense, and splice site mutations in tumor suppressor genes, were identified as driver mutations.

### 2.4 TMB Calculation

TMB was determined by counting all synonymous and nonsynonymous somatic SNVs and indels in the coding sequences. Known driver mutations were excluded to prevent inflating the mutational load. The mutation count was normalized to the size of the coding region (218 kb) that was covered by the custom panel. The TMB was calculated as the number of mutations per megabase [32].

### 2.5 Detection of CNAs

CNA analysis was conducted using ONCOCNV, employing blood DNA from enrolled patients as a reference [33]. Tumor‐ and blood‐derived BAM files were aligned to the reference genome and normalized for read depth. Normalized tumor reads were compared with the blood sample reads to identify CNAs. The CNA status was categorized as high‐level amplification (CN ≥ 8), low‐level gain (CN ≥ 4 and < 8), shallow deletion (CN ≤ 1 and > 0.5), and deep deletion (CN ≤ 0.5). For male patients, the CNA of genes on chromosome X was classified as high‐level amplification (CN ≥ 4), low‐level gain (CN ≥ 2 and CN < 4), shallow deletion (CN ≤ 0.5 and CN > 0.25), and deep deletion (CN ≤ 0.25).

### 2.6 Statistical Analysis

The chi‐square test was used to analyze the mutation frequency across the melanoma subtypes. Differences in median TMB and CNAs across melanoma subtypes were analyzed using the Mann–Whitney *U* test. Statistical significance was determined using a *p* < 0.05.

## 3 Results

### 3.1 Clinicopathological Characteristics of the Patients

This study included 104 patients: 37 with CM, 52 with AM, and 15 with MM (Table 1). Tumor samples were collected from 73 patients with primary lesions only, 10 with metastatic lesions only, and 21 with both primary and metastatic lesions. One patient (S27) with CM and eight patients (M06, M11, M15, M16, M22, M28, M32, and M62) with AM underwent drug treatment for melanoma prior to metastatic tissue sampling (Table S2). Treatments included ICIs, BRAF/MEK inhibitors, dacarbazine monotherapy or combination therapies, and interferon‐β perilesional dermal injections. Another patient (S07) with AM underwent bone marrow transplantation for lymphoma 17 years prior to melanoma resection.

**TABLE 1:** Patient characteristics.

	Cutaneous	Acral	Mucosal
N	37	52	15
Age (years)			
Median	65	73	72
Range	24–89	46–95	43–85
Gender			
Male	22	28	2
Female	15	24	13
Tumor thickness (mm)			
Median	4.9	5.0	6.5
Range	2.1–34	0.55–31	2.0–10
Stage^a^			
I	0	1	1
II	16	17	7
III	18	31	1
IV	3	3	5
Site of registration			
SMU	23	34	12
SU	14	18	3

Abbreviations: SMU, Sapporo Medical University Hospital; SU, Shinshu University Hospital.

^a^ The 8th edition of the American Joint Committee on Cancer melanoma staging system.

### 3.2 Somatic and Germline SNVs/Indels in Coding Sequences

**FIGURE 1:**
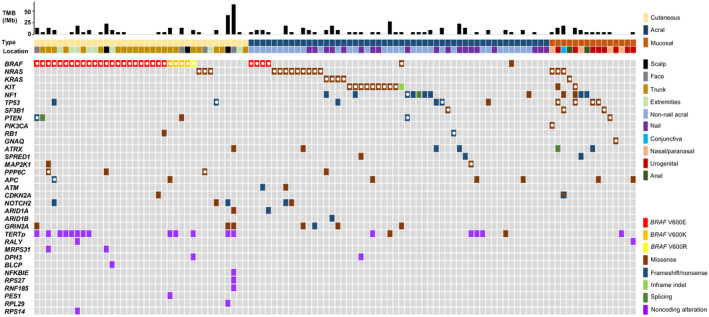
Mutation landscapes of cutaneous, acral, and mucosal melanomas. The upper panel shows tumor mutational burden (TMB). The middle panel shows the clinical details of the primary lesion type and location. The bottom panel displays an oncoplot of single‐nucleotide variants and indels. The white solid circle indicates a mutation ranked as Tiers 1–3 in the COSMIC Cancer Mutation Census. *TERTp*, *TERT* promoter mutation.

Driver mutations were detected in 32 of 37 (86%) patients with CM, 39 of 52 (75%) patients with AM, and 12 of 15 (80%) patients with MM (Figure 1 and Table S2).

In patients with CM, most driver mutations were detected in *BRAF* (28/37, 76%), of which 82% V600E, 14% V600K, and 4% V600R. *NRAS* Q61L and Q61R mutations were detected in 3 of the 37 patients (8%) and were mutually exclusive with *BRAF* mutations. Truncating *TP53* mutations were detected in two patients; one coincided with *BRAF* V600E, and the other occurred in a *BRAF*‐wild and *NRAS*‐wild tumor. Two *PTEN*, two *PPP6C*, and one *APC* mutations were detected concurrently with either *BRAF* or *N* *RAS* mutations.

The driver mutations in AM exhibited greater diversity than those in CM. *BRAF* V600E, *NRAS*, *KRAS*, and *KIT* driver mutations were detected in 4 (8%), 9 (17%), 4 (8%), and 10 (19%) of tumors, respectively, and were mutually exclusive. A *BRAF* G469R driver mutation was detected in the metastatic tissue of a patient (M16), whereas a *KIT* driver mutation was detected in the primary tumor of the same patient (detailed in a later section). *NRAS* mutations occurred at codon 61 in 6 tumors. Three *NRAS* mutations (G12R, G12D, and G13R), which were infrequent in CM, were detected. The *KIT* mutations included six L576P, one A502_Y503dup, one V560D, one K642E, and one V654A. V560 and L576 are in exon 11, whereas K642 and V654 are in exon 13. The other mutations mutually exclusive of *BRAF*, *NRAS*, *KRAS*, and *KIT* were *SF3B1*, *RB1*, *ATRX*, *SPRED1*, and *MAP2K1*. *NF1* mutations were detected in seven (13%) AMs. In three cases, *NF1* mutations co‐occurred with other driver mutations including *KRAS*, *KIT*, and *PTEN*.

*BRAF* mutations were not detected in the patients with MM. Three *NRAS* (20%), one *KRAS* (7%), two *NF1* (13%), one *PTEN* (7%), and one *GNAQ* (7%) driver mutations were detected, all of which were mutually exclusive. *NRAS* mutations included G12A, G13C, and Q61K. Two *KIT* mutations (13%) were also identified. *KIT* D816V is commonly observed in mast cell neoplasms, whereas T847M has been identified in four malignant neoplasms, including conjunctival melanoma [31]. *TP53* mutations were found in four cases (27%). Three of them were concomitant with either *NRAS*, *KIT*, or *ATRX* mutations. *SF3B1* R625 mutations, previously recognized as MM drivers in lower body sites [5], were detected in conjunctival and rectal MM. The former was concomitant with an *NRAS* mutation.

None of the patients had a family history of melanoma. No pathogenic germline variants were detected in genes associated with familial melanoma or other hereditary cancers, including *CDKN2A*, *CDK4*, *BAP1*, *PTEN*, *TERT*, *STK11*, *MITF*, *BRCA1*, *BRCA2*, or *TP53*.

### 3.3 Heterogeneity of Mutations Between Primary and Metastatic Lesions

Of the 21 patients who provided samples of both primary and metastatic lesions, 11 exhibited differing mutations (Tables 2 and S2). Patients M16 and M32 underwent perilesional dermal interferon‐β administration to the primary site before metastatic lesion sampling, whereas the other patients received no drug therapy for melanoma before sampling. In patient M05, the *RB1* nonsense mutation detected in the primary lesion was excluded from metastatic lesion analysis due to its low allele frequency (0.1%). In patient M16, the metastatic lesion lacked the *KIT* A502_Y503dup mutation but harbored the *BRAF* G469R mutation. The *BRAF* mutation was also present in the primary lesion (allele frequency, 0.09%), which indicated that the minor clone with *BRAF* G469R in the primary lesion predominated in the metastatic lesion. In patient M61, we detected the *NOTCH2* mutation in the primary lesion; however, the mutant allele frequency in the metastatic lesion fell below the mutation call threshold. Alternatively, the *TP53* mutation, which was undetected in the primary lesion, was present in the metastatic lesion. In patient S11, the *PIK3CA* mutation was exclusively detected in the metastatic lesion. The *TERT* promoter mutation was absent in the metastatic lesions of two patients (M19 and S09). Other mutations detected exclusively in one lesion were subclonal, likely representing passenger mutations.

**TABLE 2:** Cases in which different mutations were detected in primary and metastatic lesions.

Patient	Primary lesion only	Metastatic lesion only	Detected in both lesions
M05	*RB1* Q689X (14%)	nc	nc
M12	nc	*NOTCH1* C1271W (12%), *RAC1* intron (35%)	*BRAF* V600E
M16	*KIT* Y503_F504dup (15%)	*BRAF* G469R (17%)	*GRIN2A* Q1209R
M19	*TERT* promoter (46%)	nc	*BRAF* V600E
M32	*BRCA2* F851L (8%), *NTRK3* H532D (14%)	*ATM* P383R (8%)	*NRAS* Q61H, *NOTCH2* R2256H
M61	*NOTCH2* S125Efs (16%)	*TP53* D49Nfs (36%), *APC* S1465Wfs (23%)	*BRAF* V600E
S01	*HDAC9* R230Tfs (19%)	nc	*APC* N961D
S09	*TERT* promoter (6%)	nc	*MAP2K1* F53L
S11	nc	*PIK3CA* H1047L (19%), *TYR* S192Y, (78%), *BAP1* 5′‐UTR (24%)	*NRAS* G12A
S30	*PTEN* splicing (35%)	nc	*BRAF* V600E, *ATRX* L767L, *KDR* intron, *HDAC9* intron
S33	*NF1* G675R (32%)	nc	nc

*Note:* numbers in parentheses indicate variant allele frequencies.

Abbreviation: nc, not called.

### 3.4 Somatic CNAs Identified in Sequenced Genes

**FIGURE 2:**
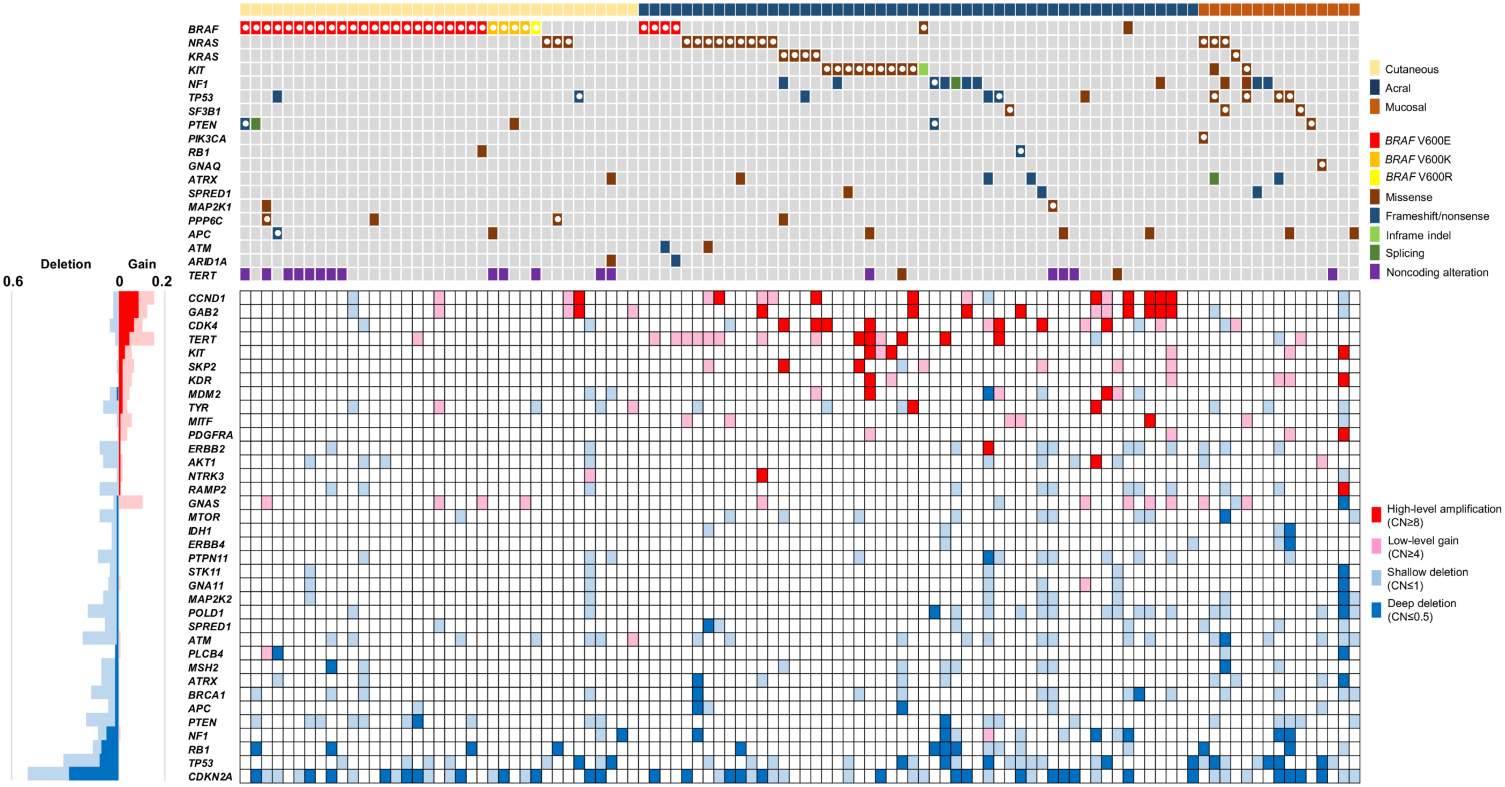
Copy number alterations in cutaneous, acral, and mucosal melanomas. The upper panel shows the melanoma subtype and principal driver mutations. The bottom panel shows copy number alterations (CNAs). The bottom left panel shows the frequency of each CNA across the cohort. Genes without high‐level amplifications or deep deletions were omitted. CN, copy number.

CNAs of well‐known oncogenes and tumor suppressor genes were frequently detected, including amplifications of *CCND1*, *GAB2*, *CDK4*, *TERT*, and *KIT* as well as deletion of *CDKN2A*, *TP53*, *RB1*, and *NF1* (Figure 2). Of the 104 tumors, 43 (41%) exhibited high‐level amplification or deep deletion of *CCND1*, *CDK4*, or *CDKN2A*. Among them, all but two CNAs were mutually exclusive.

**FIGURE 3:**
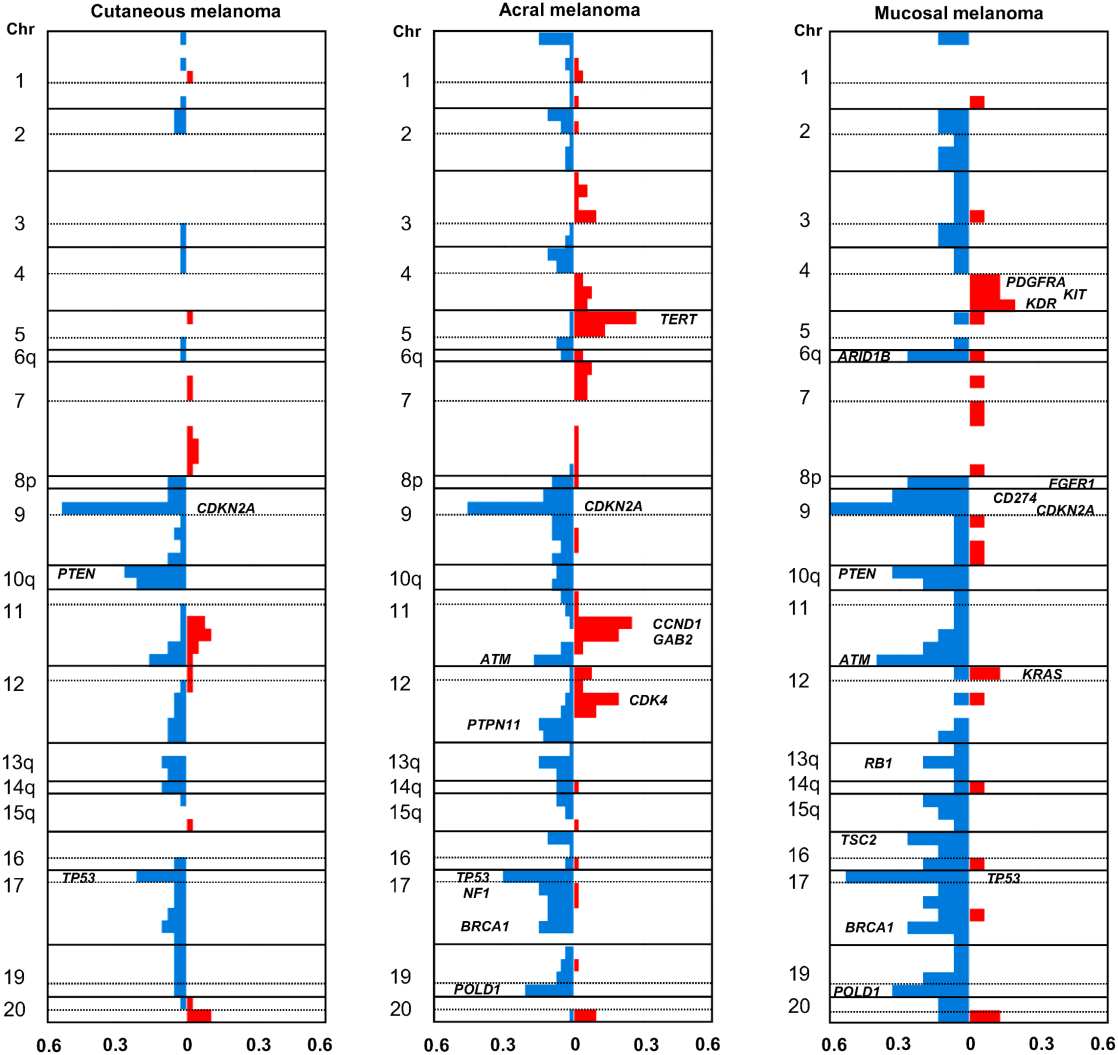
Differences in copy number alteration frequencies among cutaneous, acral, and mucosal melanomas. The frequency of copy number alterations in each gene was plotted for cutaneous, acral, and mucosal melanomas. Red bars: high‐level amplification and low‐level gain. Blue bars: shallow and deep deletions. Numbers on the left of each panel indicate the chromosome (Chr) number.

When observing CNAs across melanoma types, *CDKN2A* loss was frequent in all three (Figure 3). Loss of *TP53* occurred in all three melanoma types, more frequently in AM and MM than in CM. AM frequently exhibited gains of *TERT*, *CCND1*, *GAB2*, and *CDK4* and loss of *NF1*, *BRCA1*, and *POLD1*. MM frequently exhibited loss of *ARID1B*, *CD274*, *PTEN*, *ATM*, and *POLD1*.

**FIGURE 4:**
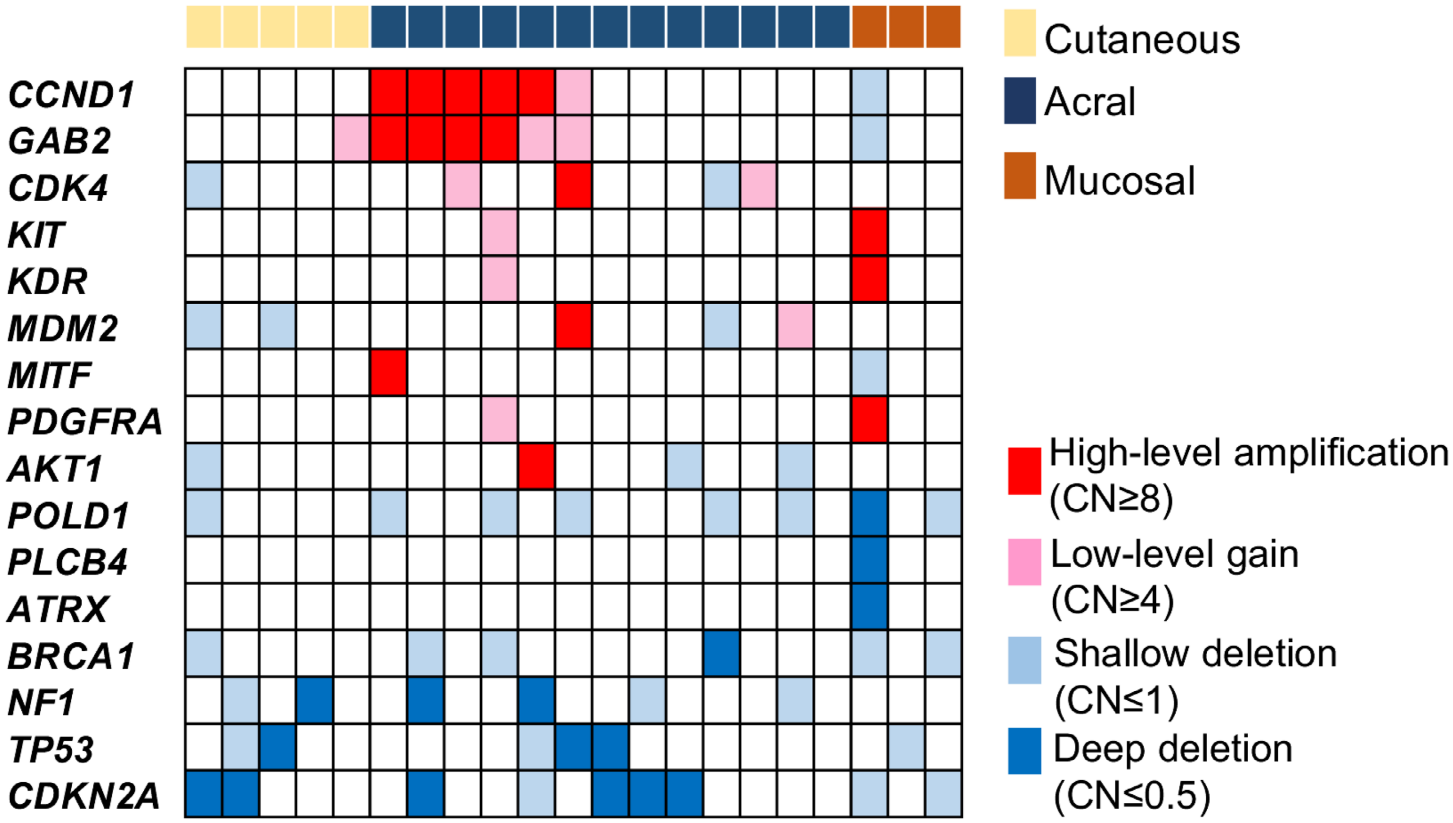
Copy number alterations in driver mutation‐negative melanoma. Copy number alterations in cases lacking driver single‐nucleotide variants or indels are depicted. CN: copy number.

Through SNV/indel analysis, no driver mutations were found in 21 patients, including 5 with CM, 13 with AM, and 3 with MM. Among these tumors, 4 CM, 10 AM, and 1 MM exhibited high‐level amplification of oncogenes and/or deep deletion of tumor suppressor genes (Figure 4). CNAs may function as driver events in these tumors. When CNAs in oncogenes and tumor suppressor genes were included as driver events alongside SNVs and indels, driver events were identified in 94% of the cases.

### 3.5 Alterations in the Upstream Noncoding Sequence

Four types of *TERT* promoter mutations that create new binding sites for the E26 transformation‐specific (ETS) family transcription factor GA‐binding protein were detected in 35% of CM (13/37), 8% of AM (4/52), and 7% of MM (1/15) (Figure 1 and Table S2). The mutation frequency in CM was lower than that in previous reports (~86%) [1, 4]. However, the mutation frequency in CM was significantly higher than that in AM or MM (CM vs. AM, *p* = 0.001; CM vs. MM, *p* = 0.03), consistent with prior studies [4]. Among previously reported alterations other than the *TERT* promoter mutation, *RALY* mutations were identified in one CM and one MM. *DPH3* mutations were identified in one CM and one AM. Other recurrent promoter mutations in *BLCAP*, *NFKBIE*, *RPS27*, *RNF185*, and *RPL29* and 5′‐untranslated region mutations in *MRPS31*, *PES1*, and *RPS14* were exclusive to CM.

### 3.6 TMB and CNA in Cutaneous, Acral, and Mucosal Melanoma

**FIGURE 5:**
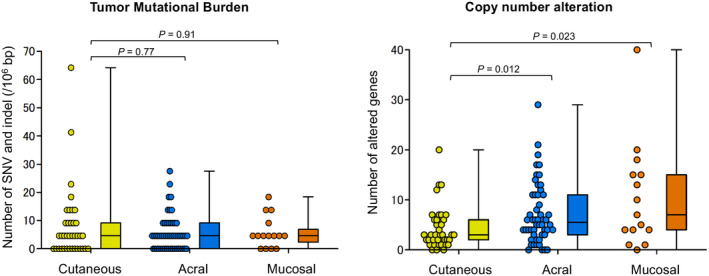
Tumor mutational burden and number of copy number alterations in each melanoma type. Tumor mutational burden and number of copy number alterations are depicted in scatter and box plots. Differences in the median values were analyzed using the Mann–Whitney *U* test.

All CM, AM, and MM exhibited median TMBs of 4.6 mutations/Mb (Figure 5). There were no statistically significant differences between CM and AM, or CM and MM. The median number of genes with CNAs was 3, 5.5, and 7 in CM, AM, and MM, respectively. AM and MM exhibited significantly higher CNAs than CM.

### 3.7 Actionable Mutations

The detected genetic alterations were analyzed for actionability using the OncoKB database [34]. Actionable mutations were categorized based on their evidence levels (Table 3). Our panel sequencing identified 58 patients (7 CM, 40 AM, and 11 MM) with potentially actionable mutations amenable to approved or off‐label drug targeting, along with 31 patients (27 CM and 4 AM) with melanoma with clinically detectable *BRAF* V600E/K mutations.

**TABLE 3:** Number of actionable mutations detected in this study.

Gene	Alteration	Evidence^a^	Drug	CM	AM	MM	Total
*BRAF*	V600E/K	1	BRAF and MEK inhibitors	27	4	0	31
	V600R	1	Vemurafenib + Cobimetinib + Atezolizumab	1	0	0	1
		2	BRAF and MEK inhibitors				
	G469R	4	PLX8394	0	1	0	1
*KIT*	L576P, V560D, K642E, and V654A	2	Imatinib	0	8	0	8
	D816, A502_Y503dup	1^b^	Imatinib, Regorafenib, Sunitinib, Ripretinib, and Avapritinib	0	1	2	3
*NRAS*	Oncogenic mutations	3A	Binimetinib	3	9	3	15
		4	Binimetinib + Ribociclib				
*MAP2K1*	Oncogenic mutations	3A	MEK inhibitors	0	1	0	1
*CDKN2A*	Deletion	4	Palbociclib, Ribociclib, and Abemaciclib	11	14	4	29
*NF1*	Oncogenic mutations	4	MEK inhibitors	0	6	2	8
*KRAS*	Oncogenic mutations	4	MEK inhibitors	0	4	1	5
	G12	4	RMC‐6236				
	G12D	4	ASP3082, MRTX‐1133				
*PTEN*	Oncogenic mutations	4	GSK2636771, AZD8186	0	1	1	2
*PIK3CA*	Oncogenic mutations	4	RLY‐2608	0	0	1	1
	H1047L	1^b^	Alpelisib + Fulvestrant				
*ARID1A*	Truncating mutations	4	PLX8394, Tazemetostat	0	1	0	1
*ATM*	Oncogenic mutations	1^b^	PARP inhibitors	0	1	2	3
*BRCA1*	Deletion	1^b^	PARP inhibitors	0	2	0	2
*ERBB2*	Amplification	1^b^	HER2 inhibitors	0	1	0	1
*PDGFRA*	Amplification	2^b^	Imatinib, Dasatinib, Regorafenib, Sunitinib, and Ripretinib	0	0	1	1
*CDK4*	Amplification	4^b^	Abemaciclib and Palbociclib	0	7	0	7
*SF3B1*	Oncogenic mutations	4^b^	H3B‐8800	0	1	2	3
*MDM2*	Amplification	4^b^	Milademetan	0	2	0	2
*Total*				42	64	19	125

^a^ OncoKB therapeutic level of evidence [34], 1: US FDA‐recognized biomarker predictive of response to an FDA‐approved drug in this indication, 2: Standard care biomarker recommended by the National Comprehensive Cancer Network or other professional guidelines predictive of response to an FDA‐approved drug in this indication, 3A: Compelling clinical evidence supports the biomarker as predictive of drug response in this indication, 4: Compelling biological evidence supports the biomarker's predictive value for drug response.

^b^ For other cancer indications. CM, cutaneous melanoma; AM, acral melanoma; MM, mucosal melanoma.

## 4 Discussion

In targeted sequencing analysis using our custom panel, we identified major driver SNV/indel mutations in 80% of cases. When considering high‐level amplification in oncogenes and deep deletion in tumor suppressor genes as driver events, they were detected in 94% of the cases. These detection rates match those of prior target, whole exome, and whole genome sequencing studies [1, 4, 5, 6, 35, 36]. This approach successfully mapped the genetic landscape of Japanese melanoma.

Surprisingly, TMB did not differ significantly between CM, AM, and MM. Previous studies in Western countries have shown that CM exhibits higher TMB and fewer structural variants and CNAs compared with AM and MM [4, 5, 6, 16]. CM was found to have an average of 49.17 mutations/Mb, 18 times higher than the 2.64 mutations/Mb observed in AM and MM [4]. Among CM subtypes, low‐CSD, high‐CSD, and desmoplastic melanoma displayed median TMBs of 15, 30, and 62 mutations/Mb, respectively [2]. The lower TMB observed in CM in this study compared to that in previous reports may be attributed to the inclusion of only one high‐CSD melanoma and the absence of desmoplastic melanoma in patients with CM. Despite these considerations, the TMB in CM remained low. Another reason could be the ethnic composition of participants. In previous studies from Western countries, most CM patients were Caucasian, whereas AM and MM cohorts included non‐Caucasians, including East Asians. Thus, TMB in non‐Caucasian patients with CM may be less characterized and potentially lower than that in Caucasian patients. Two other studies in China showed a similar trend in TMB among CM, AM, and MM, with all median TMB values being less than 10 mutations/Mb [35, 36]. Our results might indicate a broader trend: East Asians have a lower TMB in CM than Caucasians. This difference in TMB could account for the reduced efficacy of ICIs in CM among East Asians compared with Western populations [15, 17].

*BRAF* mutations were detected in 76% of CM cases, exceeding the previously reported range of 46%–64% [1, 4, 16, 18, 35, 36]. The prevalence of *NRAS* mutations in CM was only 8%, which is less than the 28%–40% found in Western studies [1, 4, 16], but aligns with the 8%–12% reported in Japan and China [18, 35, 36]. These variances may be due to racial differences and the composition of our CM cohort, which included only one high‐CSD melanoma and no desmoplastic melanomas, representing a lower incidence of these subtypes compared with previous studies. High‐CSD melanoma frequently harbors *NRAS*, *BRAF* non‐V600E, and *KIT* mutations. In contrast, desmoplastic melanoma frequently harbors *NF1* mutations [2].

The frequency of major driver mutations in AM differed from that in previous meta‐analyses, which were mainly conducted in North America and Australia (*n* = 181): [37] *BRAF* (10% vs. 21%), *NRAS* (17% vs. 14%), *KIT* (19% vs. 9%), *NF1* (13% vs. 6%), and *PTEN* (2% vs. 4%). The frequencies of *BRAF*, *NRAS*, and *KIT* mutations were consistent with prior hotspot sequencing analyses in Japan: [18] *BRAF* (10% vs. 10%), *NRAS* (17% vs. 16%), and *KIT* (19% vs. 19%). These findings likely represent the mutational profile characteristics of Japanese AM. Other notable mutations were observed in *KRAS*, *TP53*, *SF3B1*, *RB1*, *ATRX*, and *SPRED1*. *KRAS* was detected in 8% of the patients with AM in this study. The frequency was higher than in previous North American and Australian studies (0%–4%) [4, 6, 16, 38] but similar to a Chinese study (7%) [35], possibly reflecting East Asian AM characteristics. Among the remaining AMs lacking driver SNV/indel mutations, CNAs in *CCND1*, *GAB2*, *CDK4*, *TP53*, *CDKN2A*, and *BRCA1* likely served as driver events. Frequent high‐level amplification of *TERT*, rather than promoter mutations, is a typical feature of AM, as previously described [37, 38].

Major driver mutations, excluding *BRAF* mutations, were detected in the MM cohort. No *BRAF* mutations were detected in MM in either this study or in a previous Japanese study [18] (*n* = 37 in total). Among studies conducted in Western countries and China, the frequency of *BRAF* mutations ranged from 0% to 23% [4, 5, 35, 36]. These differences may stem from the small sample sizes and varied anatomical sites of the samples. For instance, *BRAF* V600E and *NF1* mutations occur more frequently in conjunctival melanomas [5, 39]. The frequency of CNAs in MM was similar to that in AM. Deep deletions of *CDKN2A* and *TP53* were frequently observed, whereas high‐level amplification of oncogenes occurred less often than in AM.

The study cohort comprised 31 patients (30%) with melanoma harboring the *BRAF* V600E/K mutation, treatable with *BRAF*/MEK inhibitors. Our panel sequencing analysis revealed additional actionable mutations in 58 patients (56%), indicating potential treatment options with commercially available drugs, excluding those in preclinical stages. However, evidence supporting these treatments varies. The *BRAF* V600R mutation, the only one with evidence level 1, is treatable with BRAF/MEK inhibitors and ICI. *KIT* mutations in exons 11 and 13 show responsiveness to tyrosine kinase inhibitors such as imatinib, with response rates between 21.4% and 66.7% (evidence level 2) [40]. Binimetinib extends progression‐free survival in *NRAS*‐mutated melanoma, but its impact on overall survival is not significant when compared to dacarbazine (evidence level 3A) [41]. The ribociclib and binimetinib combination has demonstrated effectiveness in *NRAS*‐mutated melanoma, especially in patients with additional cell cycle gene mutations (*CDKN2A*, *CDK4*, and *CCND1*), who may derive greater benefit from this therapy [42]. MEK inhibitors are successful in treating Langerhans cell histiocytosis with *MAP2K1* mutations (evidence level 3A), but their efficacy in melanoma remains uncertain [43]. Other genetic alterations with potential for targeted therapy (evidence level 4) include *BRAF* G469R, *NF1*, *KRAS*, and *PTEN*. Moreover, genetic alterations targetable in other cancers were identified in *KIT*, *PIK3CA*, *ATM*, *BRCA1*, *ERBB2*, *PDGFRA*, CDK4, and *MDM2*, some of which were reported to respond to treatments also in melanoma cases in individual case reports [44, 45].

Of the 21 cases studied, 11 exhibited notable genetic divergence between primary and metastatic tumors, highlighting clonal cancer evolution [46]. Foundational mutations such as *BRAF* V600E, *NRAS* G12A/Q61H, and M*AP2K1* F53L were prevalent across clones. In contrast, clade mutations such as *TP53* D49Nfs* and *PIK3CA* H1047L were exclusively identified in metastases, suggesting that they are later evolutionary events. Some driver mutations (e.g., *RB1* Q689X and *KIT* Y503_F504dup) were absent in metastatic samples, suggesting clonal progression without these mutations. The inherent heterogeneity of melanoma underscores the complexity of its treatment given the diverse responses and resistance patterns exhibited by distinct clones within an individual patient [47]. To effectively address this challenge, thorough genetic analyses of all tumor clones are imperative. Liquid biopsies using circulating tumor DNA (ctDNA) offer a practical approach as they noninvasively capture the genetic diversity of the tumor [48]. For future research, advanced strategies such as deep sequencing to identify subclones in primary lesions, digital PCR for ctDNA monitoring, and further mutational profiling with ctDNA to detect new somatic mutations during treatment and follow‐up are crucial for enhancing personalized treatment approaches.

This study had several limitations. First, we selected cases in which tumor samples were obtained through macrodissection. This selection process was neither sequential nor exhaustive and potentially misrepresented the frequency of genetic alterations more broadly. Second, bulk sequencing of tumor samples likely resulted in the omission of subclonal mutations with low allele frequencies. Third, structural variants and fusion genes, which are pivotal in driver mutation‐negative melanoma, were undetectable using our methodology. Additionally, new genes significant to melanoma are reported annually, and our limited gene panel sequencing cannot encompass all of them. As next‐stage research, sequential fusion gene analysis and whole genome sequencing could be considered for cases where panel sequencing does not detect drivers. Finally, the sample size of this study was small, with only 15 mucosal melanoma cases enrolled. The results obtained from the current sample size do not represent Japanese melanoma patients' genetic characteristics, especially for mucosal melanoma. One way to obtain more representative data on Japanese melanoma is by analyzing the clinical genome profiling data that have been accumulated nationally in the Center for Cancer Genomics and Advanced Therapeutics (C‐CAT) [49]. The patient cohort and mutation detection methods in C‐CAT differ from those in our study. However, comparing cohorts obtained by different methods would enhance the resolution for detecting genetic abnormalities.

In summary, the custom panel sequencing revealed numerous previously unknown genetic abnormalities in Japanese melanoma patients, enabling a comparison of the genetic landscape across different ethnicities. When stratified by subtype (CM, AM, and MM), melanoma cases exhibited mutation frequencies consistent with those reported in Western countries and other East Asian regions. However, unique genetic abnormalities in East Asians, particularly in Japanese individuals, were also identified. Notably, TMB in East Asian CM may be lower than that in Western CM, potentially affecting ICI efficacy. Additionally, the custom panel sequencing identified numerous actionable mutations. Although the evidence is currently limited, the expansion of comprehensive genetic analysis in clinical melanoma settings is expected to enhance patient access to targeted molecular therapies.

## Author Contributions

**Tokimasa Hida:** formal analysis (lead), funding acquisition (lead), investigation (lead), project administration (lead), writing – original draft (lead). **Masashi Idogawa:** formal analysis (equal), investigation (equal), writing – review and editing (equal). **Junji Kato:** resources (equal), writing – review and editing (equal). **Yukiko Kiniwa:** investigation (equal), resources (equal). **Kohei Horimoto:** resources (equal). **Sayuri Sato:** resources (equal). **Masahide Sawada:** funding acquisition (equal). **Shoichiro Tange:** formal analysis (equal), investigation (equal). **Masae Okura:** investigation (equal). **Ryuhei Okuyama:** supervision (equal). **Takashi Tokino:** supervision (equal). **Hisashi Uhara:** conceptualization (equal), funding acquisition (equal), supervision (lead), writing – review and editing (equal).

## Ethics Statement

Approval of the research protocol by an Institutional Review Board (IRB) and Informed Consent: The protocol adheres to the Declaration of Helsinki provisions. Informed consent was obtained from subjects providing tumor and normal tissue samples. This study received approval from the IRBs of Sapporo Medical University Hospital and Shinshu University Hospital (IRB‐282‐224). The remaining patients were informed about the study involving archival FFPE specimens through a website and could opt out. This study was approved by the IRB of Sapporo Medical University Hospital (IRB‐332‐192).

## Conflicts of Interest

T. H. received research grants from the Maruho Takagi Dermatology Foundation, Suhara Memorial Foundation, and ITO Foundation. H. U. received research grants from Maruho corporation, Sun Pharma corporation, Taiho Pharmaceutical corporation, and Torii Pharmaceutical corporation. The study was not designed under the responsibility of any corporations; No materials were donated/provided by any corporations. No corporations collected and analyzed the data and contributed to the interpretation of the study. All authors had full access to all of the data in the study and had final responsibility for the decision to submit for publication; M. I., J. K., Y. K., K. H., S. S., M. S., S. T., and M. O. have no conflict of interest.

## Animal Studies

The authors have nothing to report.

## Permission to Reproduce Material From Other Sources

The authors have nothing to report.

## Supporting information


**TABLE S1.** Genes included in the custom melanoma gene panel.

**TABLE S2.** All somatic single‐nucleotide variants and indels.

## Data Availability

The FASTQ files that support the findings of this study are openly available in the NBDC Human Database at https://humandbs.dbcls.jp/en/ (dataset ID JGAS000351) .
